# Review: Quality of Life in Children with Non-cystic Fibrosis Bronchiectasis

**DOI:** 10.3389/fped.2017.00084

**Published:** 2017-04-24

**Authors:** Anna Marie Nathan, Jessie Anne de Bruyne, Kah Peng Eg, Surendran Thavagnanam

**Affiliations:** ^1^Department of Pediatrics, University of Malaya, Kuala Lumpur, Malaysia; ^2^University Malaya Pediatric and Child Health Research Group, University of Malaya, Kuala Lumpur, Malaysia

**Keywords:** quality of life, child, bronchiectasis, cough, review, treatment

## Abstract

Non-cystic fibrosis bronchiectasis (NCFB) has gained renewed interest, due to its increasing health-care burden. Annual mortality statistics in England and Wales showed that under 1,000 people die from bronchiectasis each year, and this number is increasing by 3% yearly. Unfortunately, there is a severe lack of well-powered, randomized controlled trials to guide clinicians how to manage NCFB effectively. Quality-of-life (QOL) measures in NCFB are an important aspect of clinical care that has not been studied well. Commonly used disease-specific questionnaires in children with NCFB are the St George’s Respiratory Questionnaire, Short Form-36, the Leicester Cough Questionnaire, and the Parent Cough-Specific Quality of Life questionnaire (PC-QOL). Of these, only the PC-QOL can be used in young children, as it is a parent-proxy questionnaire. We reviewed pediatric studies looking at QOL in children with NCFB and cystic fibrosis. All types of airway clearance techniques appear to be safe and have no significant benefit over each other. Number of exacerbations and hospitalizations correlated with QOL scores, while symptom subscales correlated with lung function, worse QOL, frequent antibiotic requirements, and duration of regular follow-up in only one study. There was a correlation between QOL and age of diagnosis in children with primary ciliary dyskinesia. Other studies have shown no relationship between QOL scores and etiology of NCFB as well as CT changes. As for treatments, oral azithromycin and yoga have demonstrated some improvement in QOL scores. In conclusion, more studies are required to accurately determine important factors contributing to QOL.

## Importance of Quality-of-Life (QOL) Assessments in Children with Non-Cystic Fibrosis Bronchiectasis (NCFB)

Non-cystic fibrosis bronchiectasis, once called an “orphan” disease, has recently gained renewed interest. In the face of increasing burden and mortality rates in adults from bronchiectasis (1), compounded by lack of high quality research in key areas especially treatment (2), the European Multicentre Bronchiectasis Audit and Research Collaboration was set up in 2012 to promote clinical and translational research in bronchiectasis (3). Hopefully, this initiative will provide much needed information that will eventually translate to reduced mortality and improved QOL (3).

As we aim toward a holistic approach to medicine, measuring health-related QOL in patients with chronic disease is important. Patients’ general well-being, mental state as well as impact of disease on their activities of daily living is important as it will affect compliance to medical therapy and eventually morbidity and mortality (4). Furthermore, as most chronic diseases are incurable, the aim of treatment is not to burden patients with complex treatment protocols, but instead give them practical treatment regimens that will allow them to lead normal lives, both physically and mentally. Studies on QOL in children with cystic fibrosis (CF) report them having feelings of being vulnerable, losing their independence and opportunities, and being isolated and disempowered (5). This emphasizes the importance of clinicians seeing their patient beyond the disease but from the patients’ perspective. This strategy will hopefully lead to improved outcomes.

The aim of this review is to examine existing literature on the QOL in children with NCFB and its determinants.

## Tools Used to Assess QOL

Use of disease-specific questionnaires instead of generic ones has been shown to be far superior in adult respiratory disease (6–8). In adults, the St George’s Respiratory Questionnaire (SGRQ) and the Leicester cough questionnaire have been commonly used to assess QOL in patients with respiratory disease, other than asthma (9).

The SGRQ has also been used to assess QOL of adults with COPD and bronchiectasis (10). The original version was published more than two decades ago, and since then, it has been translated into more than 70 different languages. It consists of 50 questions grouped into 3 domains: symptoms, activity, and impact (11). In a recent systematic review of QOL questionnaires in patients with bronchiectasis, there was strong correlation with subjective symptoms but a weak correlation with objective assessments such as FEV_1_, radiological scoring, and exercise capacity (9). The SGRQ has also been used to assess QOL in children with bronchiectasis as young as 8 years old (12).

Recently the Quality of Life-Bronchiectasis (QOL-B) questionnaire has been validated for use in adults with bronchiectasis. It has 37 items on 8 scales: symptoms, physical, role, emotional and social functioning, vitality, health perceptions, and treatment burden. It has been shown to reflect changes in respiratory symptoms (13, 14). However, all the above questionnaires are self-administered, hence limiting its use to older children. The QOL-B has yet to use in older children.

The Parent Cough-Specific Quality of Life questionnaire (PC-QOL) is a disease-specific parent-proxy questionnaire, looking at the impact of cough on the child and parent (15). It is a 27-item questionnaire that assesses parent’s feelings and worry related to their child’s cough, i.e., psychological (11 items), physical (11 items), and social (5 items). Lower scores reflect worse QOL. It uses a 7-point Likert-type scale with a minimum important difference of 0.9 (16). In the validation study, there was good internal consistency and when compared to the generic PedsQL and other measures of cough, there was good correlation between scores. The PC-QOL was also able to demonstrate changes over time, hence confirming its clinical utility and sensitivity (17). Hence, since cough is an important symptom in NCFB, the PC-QOL is an excellent tool to assess the burden of disease in young children. There are two versions of this questionnaire, a short version and a long version. The short version consists of only 8 questions and has been validated against the original version with 27 questions in 2 different populations and has been shown to be sensitive to change in symptoms (18).

Other tools that have been used to assess parental anxiety and stress levels in parents is the Depression Anxiety Stress Scale 21 (DASS21) (19). The DASS21 is a self-reported scale with 21 items measuring three aspects of emotional distress: depression, anxiety, and stress. Participants responded to each item on a 4-point severity scale from 0 to 3. Higher scores indicate worse mental health.

A systematic review of the validity of QOL questionnaires in adults and children with bronchiectasis, excluding CF, identified 38 suitable studies. The SGRQ, Leicester Cough Questionnaire, QOL-B, and Short Form-36 were the commonest questionnaires used. There was a stronger correlation between QOL and subjective symptom outcome measures, such as dyspnea {*r* = 0.55 [95% confidence interval (CI) 0.41–0.68]} and fatigue (*r* = 0.42 [95% CI 0.23–0.58]) compared with objective measures; exercise capacity (*r* = −0.41 [95% CI −0.54 to −0.24]), FEV1% predicted (*r* = −0.31 [95% CI −0.40 to −0.23]), and extent of bronchiectasis on CT scan (*r* = 0.35 [0.03–0.61]), suggesting that QOL questionnaires assess a unique aspect of health not captured by objective clinical measures (9).

## Knowledge Gaps

Disease-specific questionnaires for use in children of different age groups with bronchiectasis are lacking. There is a specific questionnaire for children with CF but not NCFB. Perhaps with such a questionnaire and correlations between physiological tests and QOL scores may be found, and more conclusions can be made about the impact of this disease on the child. However, other reasons why symptoms may not correlate with physiological tests, such as spirometry, may be that the relationship between lung function and symptoms is not linear and that symptoms may only be seen when there is severe deterioration of lung function. Also, to understand the psychological impact of NCFB on these children, open-ended questions should be asked to allow expression of their true feelings rather than use of close-ended questions.

## Burden of Disease in Children

Kapur et al. looked at 69 children with a median (IQR) age of 7 (3.8–10.9) years old, with NCFB and the determinants of QOL and parental mental health, during stable and exacerbation states (Table 1). Other clinical parameters that were studied were, e.g., time from diagnosis, etiology, airway resistance, and extent of disease were also studied. The PC-QOL and DASS21 were completed during stable state and an exacerbation. Impairment in QOL were more likely to be reported in parents of younger children (*r* = 0.242, *p* = 0.04), but radiological extent (*p* = 0.78), baseline lung function, chronic upper airway disease, and underlying etiology did not influence the burden of disease scores (20).

**Table 1:** **Summary of studies and their findings of quality of life (QOL) in children with non-cystic fibrosis bronchiectasis (NCFB)**.

Country, year	Number	Type of study	Age (years)	Questionnaire	Findings
Australia, 2010	69	Cross-sectional	Median (IQR): 7 (3, 8, 10.9)	PC-QOL, DASS21	Parents of young children were more likely to report an impaired QOL
					Radiological extent, baseline lung function, underlying etiology, and chronic upper airway disease did not influence the burden of disease scores
Malaysia, 2014	60 (CF = 10; others = 50)	Cross-sectional	Median (range): 1.3 (0.3–11)	PC-QOL, DASS21	Mental health of parents with children with CF were better than those with NCFB
					Frequent exacerbations, frequent cough, age of diagnosis, and age of patients were not significantly associated with PC-QOL scores
Turkey, 2014	76	Case–control	11.7 (±2.6)	CDI, STAI-C, PedsQL-P, PedsQL-C	Patients did not have depression and anxiety scores significantly different from controls
					CDI and STAI-C scores negatively correlated with QOL scores
					Parents reported worse QOL in physical, psychosocial, and total areas
					Number of exacerbations and hospitalizations, FEV_1_/FVC% predicted, dyspnea, and wheezing severity were the significant factors associated with a worse QOL
					Patients reported worse physical QOL
Turkey, 2014	42	Cross-sectional	12.7 (±2.3)	SF-36, SGRQ	Symptom subscale of SGRQ correlated positively with low lung function and frequent antibiotic requirements
					Inverse correlation between SGRQ symptom scores and the duration of regular follow-up
					No correlation between SGRQ scores and current age, age at diagnosis, age at the beginning of the symptoms, height and weight *Z*-scores, etiology of NCFB, sputum microbiology, HRCT score, and socioeconomic status
UK, 2010	78 PCD	Cross-sectional		SF-36, SGRQ	Patients with the highest treatment burden had worse QOL
					Positive correlation between time since diagnosis and improvement in perceived QOL
					No correlation between scores with age or age at diagnosis

*CDI, The Child Depression Inventory; STAI-C, State-Trait Anxiety Inventories for Children; PedsQL-P, Pediatric Quality of Life Inventory Parent Version; PedsQL-C, Pediatric Quality of Life Inventory Child Version; SF-36, Short Form-36; SGRQ, St George’s Respiratory Questionnaire; PCD, primary ciliary dyskinesia; CF, cystic fibrosis; DASS21, Depression Anxiety Stress Scale 21; PC-QOL, parent-cough specific quality of life questionnaire*.

In our own study, a cross-sectional study that involved 60 children with CF and NCFB, based in a developing country found that exacerbations more than twice in the past 6 months (*z* = −0.19, *p* = 0.21), cough more than 2 days/week (*z* = −0.148, *p* = 0.18), age of diagnosis (*r* = −0.15, *p* = 0.40), or age of patients (*r* = 0.38, *p* = 0.54) were not significantly associated with PC-QOL scores (21). Mental health of parents with children with CF was better than those with NCFB (*z* = −2.32, *p* = 0.019) (21). Furthermore, in our cohort, children with CF had significant improvements both weight and BMI compared to children with NCFB: weight (CF: *z* = +2.09, *p* = 0.04 vs non-CF: *z* = +0.95, *p* = 0.37) and BMI (CF: *z* = +2.55, *p* = 0.01 vs non-CF: *z* = +1.27, *p* = 0.21). This we postulated could be due to the treatment protocols which we adhered to while treating patients with CF, but in children with NCFB, treatment protocols are based on Level 3 or Level 4 evidence and treatment is less regimented (22).

In a case–control study conducted in Turkey, they evaluated the relationship between psychological symptoms, clinical variables, and QOL in a cohort of 76 children aged 8–17 years old with NCFB. Controls were matched for age, gender, and education level of the parents with healthy children from the local community, matched. The Child Depression Inventory (CDI), State-Trait Anxiety Inventories for Children (STAI-C), Pediatric Quality of Life Inventory Parent Version, and the Pediatric Quality of Life Inventory Child Version were used. Patients did not have depression and anxiety scores significantly different from controls; however, CDI and STAI-C scores negatively correlated with QOL scores. Patients reported worse physical QOL (*z* = −2.40, *p* = 0.018) compared to controls, while parents reported worse QOL in physical (*z* = −3.00, *p* = 0.003), psychosocial (*z* = −3.46, *p* = 0.001), and total areas (*z* = −3.47, *p* = 0.001). They also found that number of exacerbations (*r* = −0.23, *p* < 0.05), hospitalizations (*r* = −0.49, *p* < 0.001), FEV_1_/FVC% predicted (*r* = 0.26, *p* < 0.05), severity of dyspnea (*r* = 0.42, *p* < 0.001), and wheezing (*r* = −0.45, *p* < 0.001) were significant factors associated with QOL (12).

Another Turkish study looked at the QOL of 42 children aged 9–18 years old with NCFB and its risk factors, using the generic Short Form-36 (SF-36) and SGRQ (23). The risk factors investigated, besides sociodemographic variables, were lung function and high-resolution CT scores. The symptom subscale of SGRQ correlated positively with low lung function (*r* = −0.417, *p* = 0.003) and frequent antibiotic requirements (*r* = 0.303, *p* = 0.035). There was an inverse correlation between SGRQ symptom scores and the duration of regular follow-up (*r* = −0.3, *p* = 0.04). There was no correlation between total SGRQ scores and current age (*r* = −0.04, *p* > 0.01), age at diagnosis (*r* = −0.09, *p* > 0.01), age when symptoms began (*r* = −0.15, *p* > 0.01), height and weight *Z*-scores, etiology of NCFB, sputum microbiology, HRCT score (*r* = 0.57, *p* > 0.01), and socioeconomic status (*r* = 0.50, *p* > 0.01) (23).

A study that looked at QOL of 78 young children and adolescents with primary ciliary dyskinesia used the SF-36 and SGRQ, which were either emailed or posted to them. Mean age of diagnosis of the patients was 9.4 years, which is quite late. Age at diagnosis correlated with all SGRQ subscales: symptoms (*r* = 0.24, *p* = 0.04), activity (*r* = 0.40, *p* = 0.001), and impacts (*r* = 0.40, *p* < 0.001), while time since diagnosis correlated with SGRQ impacts and symptom subscales [impacts (*r* = 0.46, *p* < 0.001) and symptoms (*r* = 0.24, *p* = 0.04)]. The authors postulate that diagnosis at an older age results in worse effects on symptoms, activity, and impacts, and also that mental health is worse. Reasons for the significant positive correlation between time since diagnosis and SGRQ symptoms and impact scores could be that treatment is only partially effective and/or that adherence to treatment is progressively poorer, or both. Patients became progressively became less interested in treating their disease and adherence to treatment modalities decreased (24).

## What are the Knowledge Gaps?

The evidence of QOL in children with NCFB is very limited, and there is no consistent finding in these studies. This may be because of the different questionnaires used, the small number of patients involved, and the small number of studies. Hence, conclusions about the factors that impact QOL in children with NCFB may not be totally accurate.

## Burden of Disease in Children with CF: What Can we Learn from These Studies

There are many more studies looking at the QOL of children with CF. What could we possibly learn from the data in these children?

First, in children with CF, there is a disease-specific questionnaire, i.e., The Cystic Fibrosis Questionnaire-Revised (CFQ-R), which can be used to measure health-related QOL in children, adolescents, and adults with CF. This questionnaire has 44 questions and 12 domains, including Physical Functioning, Emotional Functioning, Treatment Burden, Respiratory Symptoms, Health Perceptions, Vitality Role Functioning, and Social Functioning (25). There are four versions for different age groups, even as young as 3 years old.

A systematic review of QOL among adolescents and adults (aged > 14 years old) with CF looked at 23 studies that examined the association between sociodemographic and clinical factors with QOL (26). It found that the most commonly studied factors were FEV1% predicted, sex, body mass index (BMI), age, and pulmonary exacerbations. This review showed that male subjects reported higher Physical Functioning but lower Body Image scores compared than females. Females with CF have a survival disadvantage and that perhaps explains the correlation between female sex and lower physical functioning (27, 28). However, female adolescents/adults may be happier with a lower BMI than males. Higher BMI correlated positively with Body Image and vitality. It has been shown that malnutrition in CF has been linked with increased mortality (29). Age correlated negatively with Treatment Burden. This is possibly due to the increased complications seen in patients with CF, as they grow older and hence the increase in treatment burden. Lung function indices, i.e., FEV1% predicted was positively associated with most of the CFQ-R 14+ domains, i.e., Physical Functioning, Health Perceptions, Vitality, Treatment Burden, Respiratory Symptoms, Role Functioning except for Digestion, Social Functioning, and Emotional Functioning, and this was a consistent finding in most studies. Pulmonary exacerbations were negatively associated with multiple domains, including Respiratory Symptoms, Physical Functioning, Vitality, Body Image, Eating Disturbances, Weight, and Role Functioning (26).

In another study that looked at young adults (18–30 years old) with CF, depression was reported in 32.8% of the participants. QOL was low on Vitality and Treatment Burden, and symptoms of depression correlated with low QOL scores (30).

Pain is also an important symptom in children with chronic disease. In a study that looked at pain in adolescent CF, aged 12–20 years old, and its association with QOL and pulmonary outcomes at baseline and at 6 months, 89% of patients reported pain in the past 3 months before the survey. It was short lived and mild to moderate in severity. Pain was associated with increased pulmonary exacerbations (OR = 1.99, *p* = 0.03) and with low QOL (31).

Finally, a systematic review on the experiences and perspectives of children and adolescents, aged 4–21 years old, with CF, found that the main psychological problems faced by these patients were the following: sense of vulnerability, loss of independence and opportunities, isolation, and disempowerment. Therefore, patient-centric care and active involvement of patients in the decision-making process is key in the management of this chronic disease to ensure optimal treatment and outcome, both in QOL and clinical (26).

## Possible Treatment Determinants of QOL

### Nutrition

Nutrition and body composition are an important determinant of mortality as well as QOL scores, i.e., Body Image and Vitality (29). In children with CF, FEV_1_ had a moderate correlation with BMI; however in adults, Maximal Expiratory Pressure and Maximal Inspiratory Pressure, measures of respiratory strength, were not associated with BMI nor FEV_1_ (32, 33).

### Azithromycin

Azithromycin is a macrolide antibiotic that has been used in bronchiectasis for its anti-inflammatory action. In a systematic review of nine studies (six adult and three children), looking at the safety and efficacy of azithromycin in NCFB in QOL, there were significant improvements in total score [weighted mean difference (MD) = −5.39, 95% CI, −9.88 to −0.89, *p*-value = 0.02], impact score (weighted MD = −5.88, 95% CI, −9.05 to −2.71, *p*–value <0.001), and change of dyspnea (weighted MD = −0.47, 95% CI, −0.57 to −0.37, *p*-value <0.001) of the SGRQ. However, there was only a trend for improvement in symptom scores (weighted MD = −13.38, 95% CI, −30.62 to 3.86, *p* value = 0.13) and activity score (weighted MD = −0.79, 95% CI, −4.67 to 3.09, *p* value = 0.69) (34). In a multicentre double-blind, randomized, parallel-group, placebo-controlled trial done in indigenous Australian, Maori and Pacific Island children, where 89 children (45 administered AZT and 44 administered placebo), aged 1–8 years old were randomized to receive AZT (30 mg/kg) or placebo once a week for 12–24 months, children on AZT showed significant reduction in exacerbations (incidence ratio 0.59, 95% CI 0.35–0.71) but increased nasal carriage of AZT-resistant bacteria (35).

### Antibiotics other than Macrolides

Prolonged oral or inhaled antibiotic treatments are sometimes used to prevent exacerbations, although the evidence is limited. Nebulized antibiotics have entered the armamentarium for bacteria eradication for children with NCFB, basically extrapolating data from adults. In adults, a systematic review published in 2014 of nebulized antibiotics in NCFB identified nine studies that looked at the effect on QOL (36). While six studies showed no difference in QOL scores between controls and intervention, one study that used nebulized gentamicin for 1 year showed improvement in SGRQ scores and another that used patients with nebulized aztreonam had improved QOL-B scores compared to controls.

### Pulmonary Rehabilitation (PR)

The aim of PR is to improve ventilatory capacity and exercise tolerance and thus finally help reduce dyspnea in these patients.

In adults, a review article found that supervised outpatient PR improved incremental shuttle walk distance and disease-specific QOL but these were not sustained at 6 months. There was also no effect on cough-related QOL or psychological symptoms. PR commenced during an acute exacerbation and continued beyond discharge had no effect on exercise capacity or QOL. The frequency of exacerbations over 12 months was reduced with outpatient ET (median, 2 vs 1; *p* = 0.013), but PR initiated during an exacerbation had no impact on exacerbation frequency or mortality (37).

### Airway Clearance Techniques (ACTs)

Airway clearance is the cornerstone of treatment of NCFB. In a Cochrane review done in 2015, on ACT in bronchiectasis, looking at seven studies, of which only one included children, studies show significant improvement in QOL with no safety issues (38).

In another systematic review, looking at 35 studies, reviewing different types of ACTs, e.g., active cycle of breathing techniques, autogenic drainage, forced expiration techniques, postural drainage, high-frequency chest wall oscillation, oscillating positive expiratory pressure, and exercise or PR, ACTs appear to be safe for individuals (adults and children) with stable bronchiectasis; where there may be improvements important clinical outcomes such as sputum expectoration, selected measures of lung function, and health-related QOL. However, multicentre randomized control trials are necessary to evaluate the different techniques of ACTs, especially in children (39).

### Inhaled Steroids

Chronic neutrophilic inflammation is a feature of bronchiectasis in adults and children. A single trial in adults reported significant reduction in sputum production over 14 days in the treatment group (inhaled indomethacin) compared with the placebo group (MD −75.00 g/day; 95% CI −134.61 to −15.39). There was also a significant improvement in the Borg Dyspnea Scale score (MD −1.90, 95% CI −3.15 to −0.65) but no significant difference between groups in terms of lung function or blood indices (40).

### Other Exercises, e.g., Yoga and Singing

Yoga is a form of exercise that involves breathing, stretching, strengthening, and meditation. It focuses not only on physical fitness but also on control of breathing and stress reduction. Postulated benefits of yoga on patients with bronchiectasis patients is by aiding with airway clearance during deep-breathing exercises, strengthening of respiratory muscles, stress reduction as well as may improve sense of well-being. In a pilot study that involved 10 patients with CF with mild to moderate disease, there was significant improvement in the mean (SD) CFQ-R respiratory domain score from screening to end of study (67.9 [11.4] to 82.1 [9.9], *p* = 0.04). However, there was no significant change in the other domain scores: the self-reported respiratory symptom score, FEV_1_, weight, or Ease of Breathing Score. No studies have been done in NCFB (41).

Singing is also another non-medical therapy that may improve lung function as well as QOL. In a small study, involving 40 children with cystic fibrosis, half were subjected to singing lessons and vocal training exercises while the other half, i.e., control group participated in non-physical activities of their choice. After 2 weeks of therapy (8 lessons) significant improvements in Maximal Expiratory Pressure were seen. Both groups had some improvments in the various components of QOL (42).

## What are the Knowledge Gaps?

Much of the data presented here involve either children with CF or adults with NCFB, especially the impact of treatment strategies on QOL. Double-blind randomized controlled trials on different treatment strategies on children with NCFB, and its effect on QOL, are lacking. There are no data on the effect of nutrition on QOL. Effect of PR, nebulized or oral antibiotics, and yoga and other exercises have not been investigated in children with NCFB.

## Conclusion

Currently, there are only five pediatric studies that specifically look at QOL in children with NCFB. QOL scores correlate more frequently with subjective measures such as symptoms rather than objective measures such as lung function. PC-QOL scores reflected change of symptoms and parents of younger children were more likely to report an impaired QOL, but radiological extent, baseline lung function, and etiology did not affect the scores. Mental health of parents with children with CF was better than parents of children with NCFB. Patients with NCFB were not more depressed or anxious but self-reported worse physical disability, compared to controls. Number of exacerbations and hospitalizations correlated with QOL scores in one study, while symptom subscales correlated with the following: lung function, worse QOL, frequent antibiotic requirements, and duration of regular follow-up. None of the studies showed a correlation between QOL scores and age at diagnosis, etiology of NCFB, and CT changes. In children with primary ciliary dyskinesia, a late diagnosis correlated with poorer QOL. In children with CF, lung function, pulmonary exacerbations, and BMI affected QOL scores. As for treatments, oral azithromycin, nebulized antibiotics, all forms of ACT, PR, and other exercises such as yoga had demonstrated some improvement in QOL scores.

## Author Contributions

AN and JB conceived and designed the study. AN, KE, and JB analyzed the data and wrote and edited the manuscript. ST edited the manuscript.

## Conflict of Interest Statement

The authors declare that the research was conducted in the absence of any commercial or financial relationships that could be construed as a potential conflict of interest.

## Appendix

### Key Concepts

- St George’s Respiratory Questionnaire, Short Form-36, the Leicester Cough Questionnaire, and the Parent Cough-Specific Quality of Life questionnaire (PC-QOL) can be used to assess quality of life (QOL) in children with bronchiectasis.
- Only the PC-QOL can be used in young children as it is a parent-proxy questionnaire.
- Number of exacerbations and hospitalizations correlated with QOL scores, while symptom subscales correlated with lung function, worse QOL, frequent antibiotic requirements, and duration of regular follow-up.
- Age of diagnosis was shown for the first time in patients with primary ciliary dyskinesia. None of the studies showed a correlation between QOL scores and etiology of non-cystic fibrosis bronchiectasis and CT changes.
- As for treatments, oral azithromycin and yoga have demonstrated some improvement in QOL scores albeit the data come from single studies or children with cystic fibrosis. All types of airway clearance techniques appear to be safe and have no significant benefit over each other.
